# Economic and Managerial Analysis of Effective Managerial Strategies on Prevention from Ketosis in Transition Period in Shahroud Commercial Dairy Farms

**DOI:** 10.5402/2011/605179

**Published:** 2011-12-27

**Authors:** H. Kashfi, A. R. Yazdani, M. Latifi, F. Shirani Bidabadi

**Affiliations:** ^1^Department of Animal Science, Gorgan University of Agricultural Sciences and Natural Resources, Gorgan, Iran; ^2^Central Veterinary Medicine Laboratary, Shahroud, Iran; ^3^Department of Agriculture Economic, Gorgan University of Agricultural Sciences and Natural Resources, Gorgan, Iran

## Abstract

The purpose of this research is to study any effects of managerial strategies on prevention of ketosis metabolic disorder in transition period in Shahroud commercial dairy farms. For this purpose, a questionnaire was prepared in order to obtain required information about the performance of these managerial strategies, performance costs, involvement situation with disorders relying upon clinical signs and treatment and health records, producing and economic situation, and fertility rate and its costs. The considered managerial guidelines include body condition score management or type evaluation in transition period, increase in dry matter intake close to parturition, using propylene glycol, using niacin, and high-quality feeding (the importance of feed quality) in transition period. Finally and upon arrangement of data, it was possible to study any effects of mentioned managerial strategies on related variants through multiple linear regressions. Furthermore, in order to study any relation among variables, we considered Pearson correlation coefficients as well. Finally, it was revealed that any application of managerial strategies for prevention from Ketosis in transition period has a significant effect in betterment of managerial and economic parameters.

## 1. Introduction

The transition period, from 3 wk before to 3 wk after parturition, is critically important to health, production, and profitability of dairy cows. Most health disorders occur during this time. There are lots of serious changes for dairy cows in transition period from the end of pregnancy up to the beginning of lactation period. This period was really important for many years and for a lot of researchers due the effects on the health, production, and profitability by focusing on nutrition and management of dairy cows Drackley [1]. Great number of involved cows and medical costs will reveal the economic importance of this disease. Ketosis is randomly fatal and has a little number of death and further damages “Herdt and Gerloff [2]”.

Both clinical and subclinical forms of ketosis are accompanied with a reduction in production, reduction of milk protein, and a reduction in fertility level (postponing of estrus, reduction of pregnancy at the first artificial inbreeding and increasing the calving interval). There is an increase in suffering of ovary cysts and metritis due to ketosis Radostits et al., [3]. The effective managerial strategies have been pointed out in different resources for preventing of metabolic disorders in transition period especially out of ketosis. It is in a way that these strategies will introduce simple and low-cost methods for prevention from metabolic disorders and further consequences. For instance, in a research published by Sauer et al. [4] about the effect of propylene glycol, it was specified that such a compound is recommended for treatment and prevention of ketosis. Propylene glycol will increase the plasma concentration of glucose, and insulin may reduce plasma concentration of BHBA and NEFA and betterment of metabolic parameters in transition dairy cows. It has been specified that glucogenic property of propylene glycol may reduce any negative effects of consuming rate of feeding and negative equilibrium of energy and ketosis incidence rate Studer et al. [5]; Formigoni et al. [6].

Regarding the effect of niacin in prevention from ketosis, Fronk et al. [7] stated that any effects of niacin in reduction of ketosis rate by its metabolic capacity for stopping NEFA mobilization is related to fat content and increase of blood glucose concentration although there is not a specific reason for such an increase as well.

Regarding Body Condition Score management as an effective strategy in ketosis prevention, Treacher et al. [8] concluded that cows with higher BCS will experience more serious negative energy balance than those with lower BCS. As a result, they may start any weight gain and BCS after parturition with more delay which is a reason that cows with a BCS more than 3.5 are more threatened for ketosis development. In other researches published by Young and Smith [9], it was confirmed that any benefit from niacin complementary in transition period may cause an increase in milk production and also milk fat a reduction of BHBA serum concentration Grummer [10]; Minor et al. [11].

Linn and Otterby [12]; Mahana [13] studied any effective managerial strategies in increasing of feeding after parturition and stated that leading cows towards feed barn may encourage them for more feeding. It is especially important when there is little feeding frequency. Sometimes it is necessary to take slowly any cows in relaxation mode and lead them towards manger. In addition and considering the importance of BCS, Markusfeld et al. [14] stated that cows with higher rate of BCS in parturition use lower dry matter intake accompanied with a weight reduction and higher body score at early lactation in comparison with cows with lower BCS. In a research published by Duffield [15], reducing the consumption intake immediately before parturition was stated as the most important reason of serious development of negative energy balance. Gillund et al. [16] stated that body condition score more than 3.5 at parturition is related with ketosis incidence rate increase. A cow with a BCS more than 3.5 has a greater chance for infecting with ketosis at parturition in comparison with a cow with a BCS lower than 3.25. In a report published by Holcomb et al. [17], it has been recommended to all dairy rearers to do anything in order to increase dry matter intake (DMI) through a period close up to parturition. This will cause the cow to use more feed after parturition and reduce any suffering from ketosis and other metabolic disorders. In a research published by Goff and Horst [18] it was stated that benefiting from liquid propylene glycol for first two days after parturition may reduce the plasma NEFA concentration and increasing the milk production in early lactation.

Also through a report published by Duffiled [15], it was revealed that feeding one liter of propylene glycol for 10 days before parturition will reduce ketosis and liver fat syndrome significantly. The real reason of which is an increase of plasma glucose concentration and insulin and reducing of plasma BHBA and NEFA as well.

Pickett et al. [19] confirmed in a research that metabolic status of dairy cows will be treated by the use of propylene glycol. In another research made by Hoedemaker et al. [20], two groups of control and users of propylene glycol are compared from IGF-I concentration and it was revealed that IGF-I concentration was so much high significantly in receiving group which is a sign of better performance of immune system at parturition and after that. Bobe et al. [21] proposed that a mixture of oral water and 1 kg of glycerol plus 1 liter of propylene glycol or 100 gr of sodium propionate per day is so much effective in treatment of ketosis.

Overton and Waldron [22]; Weiss and Gonzalo [23] introduced an effective method for prevention of ketosis by applying of niacin supplementary and simultaneous application of it with glucose precursors such as propylene glycol and sodium propionate. In another study by Tamizrad and Kardoodi [24], it was revealed that benefiting from niacin has no more effects in increasing of milk fat, protein, and dry matter without solid fat. But it may cause a reduction in incidence rate of metabolic disorders like ketosis and liver fat syndrome and also increasing the economic life of dairy cows. Also, they stated in the same research that niacin is effective in lipid metabolism even if the lipid metabolism is under the effect of hormone regulation. Didarkhah and Jamili [25] specified in a research in 2008 that used niacin supplementary in dairy cows has no more effects in milk production, average percentage of milk protein, lactose, and milk fat but has a significant effect on glucose rate, triglycerides, and plasma BHBA.

Ghorbani and Asadi Alamuti [26] stated that roughage foods and other fiber sources in the share have reduced energy rate and then increased probabilities of ketosis problems of the animal. On the other hand, any lack of roughage foods or effective fiber may cause subacute rumen acidosis and lack of energy reception.

Regarding the importance of feed quality, Mohebbi [27] stated that when cows are in negative energy balance they could not eat as enough as possible; therefore, it is necessary to use high-quality feeds for them in order to have an increase in nutrient density without any recommendation of nonsuitable feeds like wheat straw for any shares after parturition. Although it is possible to use a little of mentioned factors in preparturition period, nonsuitable raw materials in the considered shares may cause serious negative energy balance and this may reduce the density of nutrition in the shares due to their lack of digestibility. Any consumption of nonsuitable silos may lead to ketosis problem. These silos are not palatable due to having butyric acid and may reduce any feed consumption.

## 2. Material and Methods

The present research has been prepared within a time interval from January 2011 up to April 2011 for industrial herds of dairy cows at Shahroud city. There was a questionnaire for required data collection including different questions about 50 herds and analyzed accordingly. The mentioned questionnaire had some information about managerial strategies in first part for management cows in transition period to prevention from ketosis metabolic disorder including body condition score management (BCS), encouraging for consuming more dry matter (consuming dry matter management), using supplementary of propylene glycol, using niacin, and benefiting from high-quality feeding in transition period (the importance of feed quality). Also the performance costs have been measured in the same art. Next part belongs to relevant information about ketosis incidence rate with relying upon clinical signs and therapeutic and health records and also treatment costs for each head. The next part is related to production and economic information including average production parameters per head and also gross income of milk sale per head in lactation cycle. The final part of questionnaire belongs to reproduction records, average fertility, and its costs. Upon data arrangement, we had an analysis through multiple linear regression (MLR) and Statistical Software (SAS) 9.1. Also, we could calculate correlation of effective managerial strategies and related variants through a correlation method as follows:

(1) $$\begin{array}{c} Y_{1}=\beta_{01}+\beta_{11}X_{1}+\cdots +\beta_{q1}X_{a}+\varepsilon_{1},\\ Y_{2}=\beta_{02}+\beta_{12}X_{1}+\cdots +\beta_{q2}X_{q}+\varepsilon_{2},\\ \vdots \\ Y_{p}=\beta_{0p}+\beta_{1p}X_{1}+\cdots +\beta_{qp}X_{q}+\varepsilon_{p},\\ \rho_{x,y}=\frac{\mathrm{cov}\left(x,y\right)}{\sqrt{\left(\text{var}\;x\right)\left(\text{var}\;y\right)}}=\frac{\sigma_{xy}}{\sigma_{x}\sigma_{y}}. \end{array}$$

## 3. Results and Discussion

### 3.1. BCS

This managerial strategy includes the body condition score control especially before parturition in a way that it is possible to make ready a cow with suitable BCS for parturition.

The obtained results out of analysis of variance show that body condition score management has a positive and significant effect on incidence rate, ketosis treatment costs (*P* < 0.01), production average, gross income of milk sale, and average fertility (*P* < 0.05). But it has no more effects on fertility costs and artificial inbreeding from statistical view point (*P* > 0.05) (Table 1). The mentioned results are in compliance with findings of Gillund et al. [16] who have stated that cows with higher BCS show higher ketosis rate than others with lower BCS (%2.7 against %1.6). Also, they are in compliance with the results out of the researches made by Treacher et al. [8] and Markusfeld et al. [14] as stated that high rate of BCS at parturition is related to high rate of ketosis dangers.

The obtained results out of average comparison show that there is not a significant relation from incidence rate between different groups (*P* > 0.05). There was not any significant difference between treatments from treatment costs point of view (*P* > 0.05). Regarding the gross income, it is possible to say that there is not a significant difference among experimental treatments (*P* > 0.05). Also, there was not a significant difference between average fertility and artificial inbreeding (*P* > 0.01) (Table 1).

Following is relevant study of correlation coefficients between BCS management and other managerial and economic indices (Table 3).

- We calculated any relation between BCS Management and ketosis incidence rate *r* = −0.60 which was a positive effect of applied BCS in reducing of ketosis incidence rate. This means that any increase in application of BCS management may cause a reduction in ketosis incidence rate.
- Any relation between BCS and ketosis treatment costs. As it is expected any reduction in ketosis incidence rate in herds may cause required therapeutic costs. Therefore, with regard to the effect of BCS in reduction of ketosis rate, it is concluded that BCS management may cause a reduction in therapeutic costs. Correlation coefficient for such a relation is *r* = −0.54.
- Any relation between BCS and average production. We calculated the correlation coefficient of BCS relation and average production equal to *r* = 0.31 which is a sign of positive effect of this managerial strategy on milk production average in herd.
- We evaluated and found out a positive relation between BCS management and gross income (*r* = 0.32). It is possible to evaluate this relation as a direct relation of BCS and average production and also major role of average production in calculation of gross income in this research. As a result, it is expected to have betterment in gross income upon increasing the application of this managerial strategy.
- Any relation between BCS and average fertility. In this part, we found out a positive Pearson correlation coefficient (*r* = 0.28), although this relation was now so much specific like other variants but it was considerable as well.
- Any relation between BCS and fertility costs. Regarding the positive relation of BCS in betterment of fertility average, it is expected that by further effects on breeding rate, BCS may cause a decrease fertility cost and artificial inbreeding.

### 3.2. Increasing of Dry Matter Consumption Close to Parturition (Feeding Management)

Upon a consideration of the results out of the variance analysis, it was specified that any increase in consuming dry matter at parturition time and some days in prior will have a positive and significant effect on managerial, producing, and economic indices. As a result, any application of this strategy will have a significant effect on ketosis incidence rate, treatment costs, production average, and gross income (*P* < 0.01). But it has not a statistical significant effect on average fertility and fertility costs and artificial inbreeding (*P* > 0.05) (Table 1). These results are in compliance with the findings out of recent researches at Aiwa and Wisconsin University about the importance of feeding at parturition and liver fat syndrome and ketosis. Also according to the results of Duffield [15], it is possible to explain that any decrease in dry matter intake immediately before parturition is the most important reason of serious development of negative energy balance. Also, the results of the research made by Holcomb et al. [17] recommend the cattle owners that they should do anything in order to increase dry matter intake through close-up period. This may cause cow to have more intake after parturition. Also, it may reduce any ketosis incidence and other metabolic disorders as well.

The results out of a comparison in averages in current research show that there is a significant difference between these two groups (executive group and a group with lack of attention) to Ketosis incidence rate from statistical point of view (*P* < 0.05). By comparing the averages, there is not a significant difference between the groups from therapeutic costs (*P* > 0.05). Although there are some differences for average production rates, gross income from milk sale, average fertility, and fertility costs, and artificial inbreeding, but it was not so much significant from statistical viewpoint (*P* > 0.05) (Table 1).

The results out of Pearson correlation coefficients show the following relations among different variables (Table 3).

- In order to study any relation between intake rate variable increase and ketosis incidence rate with calculated correlation coefficient, *r* = −0.67 was a sign of its effect in reducing of ketosis incidence rate.
- Correlation coefficient for dry matter intake and average production, *r* = 0.43 was calculated as a sign of the importance of this parameter in milk production rate.
- Any relation between dry matter intake and gross income and considering the effect of this variable on production rate and direct relation of these two factors have been calculated by *r* = 0.42.
- Any relation between fertility costs and current variable have been calculated by a negative digit (*r* = −0.15) as a sign of converse relation between these two factors with independent increase, dependent factor (relevant costs of inbreeding, and fertility). Also *r* = 0.25 is the correlation coefficient for dry matter intake and average fertility.
- Any relation between intake rate and treatment costs is a negative correlation. (*r* = −0.55) is a sign of increasing the intake rate before parturition which may cause a reduction in relevant treatment costs of ketosis.

### 3.3. Application of Propylene Glycol Supplementary

The results of variance analysis of this variable show that its application had a positive effect in betterment of managerial and economic parameters like average production, incidence rate, treatment costs, average production, gross income, average fertility, and fertility costs (*P* < 0.05) (Table 1). These results are in compliance with relevant findings out of Formigoni et al. [6] and Laranjade da Fonseca et al. [28] who stated that any application of propylene glycol may cause betterment in metabolic parameters.

Also after obtaining the mentioned results, Studer et al. [5] and Formigoni et al. [6] specified that glucogenic property of propylene glycol may cause a negative effect in intake rate and negative balance of energy on ketosis incidence rate. In a research made by Stokes and Goff [29], it was revealed that feeding of propylene glycol at parturition time may cause an increase in milk production. These findings are in compliance with the results of current research.

The results of comparing the average show that there is a significant difference between compared groups from the point of view of incidence rate and ketosis treatment costs (*P* < 0.05). Regarding both variables of average production and gross income, there is a significant difference between both mentioned groups from the point of view of average properties (*P* < 0.05). Any comparison of the average of both groups did not prove any significant differences between fertility average and fertility costs (*P* > 0.05) (Table 1).

Also, we obtained the following results after calculating the correlation coefficients in this research (Table 3).

- There was a considerable relation between using of propylene glycol and ketosis incidence rate in a way that correlation coefficient for these two variants was *r* = −0.71. This means that applying of propylene glycol may cause a considerable reduction in ketosis incidence rate.
- Regarding the positive effect of prevention, there was a considerable relation between application of propylene glycol and treatment costs (*r* = −0.73) in a way that cased a reduction in ketosis treatment costs.
- We calculated correlation coefficient for production average and applying of propylene glycol that means a performance increase due to benefiting from this eatable compound and in compliance with the findings of researches made by Stock and Goff. The correlation coefficients for gross income, average fertility, and fertility costs were, respectively, *r* = 0.67, *r* = 0.58, and *r* = −0.49 which may show the effect of propylene glycol on mentioned factors.

### 3.4. Benefiting from Niacin Supplement

Upon variance analysis, the following average comparisons and calculations were applied accompanied with calculation of correlation coefficients (Table 1).

The findings out of variance analysis show that niacin application may have a positive effect on producing, economic, and managerial parameters such as average production, gross income, ketosis rate, treatment costs, and generation parameters (fertility average and its costs) (*P* < 0.01). The results obtained by Bartlett et al. [30] may confirm any effective usage of niacin in reduction of ketosis occurrence rate. Also these results are in compliance with the findings of Young and Smith [9]; Grummer [10] and Minor et al. [11] who stated that niacin will cause a betterment and increase of milk production and increasing of milk fat. Niacin application and its simultaneous usage with glucose precursor have been introduced in other researches made by Overton and Waldron [22] and Weis and Gonzalo [23]. Also in a research made by Tamizrad and Karkoodi [24], it was stated that any usage of niacin may cause a reduction in harmful effects of energy negative balance and may cause a reduction in occurrence rate of metabolic disorders such as ketosis and fat liver syndrome and increase of economic life time of dairy cows. Furthermore, the results of this research is in contrast with the findings of a research made by Didarkhah and Jamili [25] who have stated that niacin has no more effects on milk production, average percentage of milk protein, lactose, and milk fat. The obtained results out of a comparison between the averages revealed that there is a significant relation between experiment groups from occurrence rate variants and treatment costs (*P* < 0.05). But there is not a significant difference between the mentioned groups from the viewpoints of production average, gross income, average fertility, and fertility costs (*P* > 0.05). Calculation of Pearson correlation coefficients (Table 3) show that there is a significant relation between ketosis rate and applying of niacin (*r* = −0.74). Also there is a negative correlation and significant relation between niacin application and treatment costs (*r* = −0.79). Correlation coefficients between niacin application and average production, gross income of milk sale, fertility average, and fertility costs were, respectively, equal to 0.60, 0.60, 0.64, and −0.43.

### 3.5. Benefiting from High-Quality Feed in Transition Period: The Importance of Feed Quality

The real purpose of this managerial strategy was in fact increasing the appetite of cow for consuming more dry matter. It is possible to say that this strategy intends to upgrade consuming feed rate in transition period along with more benefits in production and better functions.

The obtained results out of regression and variance analysis revealed that there is a positive relation between performing this strategy and other related variants (Table 1). This means that it will make better these variables, Therefore, applying this strategy may have a positive effect in betterment of ketosis rate, treatment costs, production average, gross income, fertility average (*P* < 0.01), and fertility costs (*P* < 0.05). These findings are in compliance with the results of different researches made by Mohebbi [27] who stated that it is necessary to use highest quality of feed for the cows in transition period because of their negative energy balance in order to increase the compress of nutrition materials in share volume and also the results of Rabelo et al. [31] and Keady et al. [32] who stated that we should prevent from feeding animals with high NDF feeds not to fill rumen at the beginning of parturition; therefore, by reducing this condition it is possible to make an increase in feeding.

The obtained results out of comparing the averages show that there is a significant difference between occurrence rate parameter at probable level of 0.05 of both groups (*P* < 0.05). There was not a significant difference between both groups from statistical viewpoint and for treatment costs, average production, gross income, average fertility, and fertility costs (*P* > 0.05) (Table 1).

Also the correlation coefficient show that there is a considerable relation between quality of feeding and occurrence rate (*r* = −0.73). Furthermore, we calculated any relation between feed quality with treatment costs *r* = −0.62, production average *r* = 0.48, gross income *r* = 0.47, average fertility *r* = 0.38, and fertility costs *r* = −0.29.

### 3.6. The Effect of Application of Cost Effective-Managerial Strategies on Prevention from Ketosis on Managerial/Economic and Producing Parameters

This variable includes a set of costs imposed on a cattleman for performing all above-mentioned factors (effective managerial strategies on prevention from ketosis). In this part, we will consider any relation as an independent variable with other related variants. The obtained results of regression performance and variance analysis (Table 2) for this variant revealed that any increase of performance costs of managerial strategies may significantly make better all economic-producing parameters (Ketosis rate, treatment costs, production average, gross income, fertility average, and fertility costs) (*P* < 0.01). This means that any increase of costs resulted from applying of managerial strategies are effective in prevention from ketosis accompanied with betterment of functions of producing units and also upgrading of managerial and economic parameters. Generally, these strategies are reasonable from economic viewpoints.

The results of average comparisons show that there are significant differences between the averages from various points of view of ketosis occurrence rate, treatment costs, production average, gross income, and average fertility (*P* < 0.05). But from statistical viewpoint, there are not significant differences among mentioned groups as well (*P* > 0.05) (Table 2).

We considered correlation coefficient for measuring any relation between performance costs of managerial strategies and other managerial and economic indices with the following results (Table 3).

- Correlation coefficient for performance costs of strategies and ketosis rate was *r* = −0.80. This means that any increase in mentioned costs in strategies performance will reduce the occurrence rate of ketosis in herds.
- Correlation coefficient for performance costs of managerial strategies and ketosis treatment costs was *r* = −0.82. As it was explained that parameter would reveal its effect on treatment costs by reducing the occurrence rate of ketosis.
- We calculated any relation between performance costs of strategies and production average as *r* = 0.72 which is a sign of its positive effect on production.
- Correlation coefficient was calculated for gross income, fertility average, and inbreeding costs and fertility, respectively, as 0.71, 0.70, and −0.54.

### 3.7. The Effect of Performance Percentage of Effective Managerial Strategies on Ketosis Prevention on Economic and Managerial Parameters

Calculation of this parameter is on percentage basis for total effective managerial strategies on ketosis prevention and as a sign of performance level of it for preventing this metabolic disorder in transition period. Regression results and analysis of variance (Table 2) show that the percentage of managerial strategies has a significant effect on betterment of economic, managerial, and producing parameters (*P* < 0.01). It is in a way that any percentage increase of these strategies may cause betterment in managerial and economic conditions of training units.

The results out of average comparisons among different groups effective on ketosis prevention show that considered groups had significant differences about the following variants like: occurrence rate (*P* < 0.01), treatment costs (*P* < 0.05), average production, and gross income (*P* < 0.05). But they did not have any statistical difference for fertility average and fertility costs variants (*P* > 0.05).

The results of correlation coefficients show that there is a close relationship between performance percentage of managerial strategies and ketosis occurrence rate (*r* = −0.98). Therefore, any increase in performing of managerial strategies has a considerable effect in prevention from ketosis as well. Also, these results confirm a positive relation of performance percentage of managerial strategies on treatment costs (*r* = −0.92). Correlation coefficient for above-mentioned variable for average production was *r* = 0.71, and for gross income was *r* = 0.71, for average fertility *r* = 0.62 and for fertility costs *r* = −0.44. As it is obvious in Tables 2 and 3 any application of high-quality feeds have the most positive effect on reducing of occurrence rate of ketosis (*r* = −0.  80). Then, we have niacin application (*r* = −0.74), propylene glycol (*r* = −0.71), and increasing dry matter consumption (*r* = −0.67), respectively, with the highest rate of relation with this variable. Also regarding any relation with treatment costs, using niacin (*r* = −0.79) and propylene glycol (*r* = −0.73) have, respectively, highest rate of relation as well. Regarding the average production and gross income they have, respectively, highest rate of relation with propylene glycol (*r* = 0.67), niacin (*r* = 0.60), benefiting from highest-quality feed (*r* = 0.48), and propylene glycol supplement (*r* = 0.58).

Regarding the fertility costs and its relation with managerial strategies, there was exactly a contrast condition. It means that there is a more relation between application rate of propylene glycol (*r* = −0.49) and niacin (*r* = −0.43) accordingly.

## Figures and Tables

**Table 1:** The effect of effective managerial strategies on ketosis and managerial/economic indices.

Economic and management indices	Management strategies in transition period effective on ketosis prevention									
	BCS management		Feed intake management		Propylene glycol		Niacin		Feed quality	
	*F*-Test	*t*-Test	*F*-Test	*t*-Test	*F*-Test	*t*-Test	*F*-Test	*t*-Test	*F*-Test	*t*-Test
Incidence rate of ketosis	27.98**	5.29^ns^	41.13**	−6.41*	50.39**	−7.10*	59.23**	−7.70*	56.94**	−7.55*
Treatment cost	20.26**	4.5^ns^	20.83**	−4.56^ns^	56.46**	−7.51*	82.25**	−9.70*	30.79**	−5.55^ns^
Average production	5.35*	2.31^ns^	11.19**	3.35^ns^	40.37**	6.35*	28.43**	5.33^ns^	14.89**	3.86^ns^
Gross income	5.64*	2.38^ns^	10.53**	3.25^ns^	40.44**	6.36*	27.93**	5.28^ns^	14.28**	3.78^ns^
Average fertility	4.26*	2.06^ns^	3.31^ns^	1.82^ns^	24.99**	5.00 ^ns^	33.42**	5.78^ns^	8.43**	2.90^ns^
Fertility costs	1.23^ns^	−1.11^ns^	1.04^ns^	−1.02^ns^	15.34**	−3.92 ^ns^	10.90**	−3.30^ns^	4.67*	−2.16^ns^

**Significant difference (*P* < 0.01).

*Significant difference (*P* < 0.05).

^ns^Nonsignificant differences (*P* > 0.05).

**Table 2:** The effect of performance costs and performance percentage of effective managerial strategies on ketosis.

Management and economic indices	Performance cost		Performance costs	
	*F*-Test	*t*-Test	*F*-Test	*t*-Test
Ketosis rate	86.75**	−9.31*	1186.94**	−34.45**
Treatment cost	100.73**	−10.04*	277.01**	−16.64*
Average production	51.74**	7.19*	51.38**	7.17*
Gross income	51.33**	7.16*	50.39**	7.10*
Average fertility	46.64**	6.83*	30.11**	5.49^ns^
Fertility costs	20.79**	−4.54^ns^	11.68**	−3.42^ns^

**Significant difference (*P* < 0.01).

*Significant difference (*P* < 0.05).

^ns^Non significant differences (*P* > 0.05).

**Table 3:** The correlation coefficients of effective managerial strategies on prevention from ketosis and other managerial and economic indices.

	Ketosis rate	Treatment cost	Average production	Gross income	Average fertility	Fertility cost
BCS management	−0.60	0.54	0.31	0.32	0.28	−0.15
Increase DMI	−0.67	−0.55	0.43	0.42	0.25	−0.14
Propylene glycol	−0.71	−0.73	0.67	0.67	0.58	−0.49
Niacin	−0.74	−0.79	0.60	0.60	0.64	−0.43
Feed quality	−0.80	−0.62	0.48	0.47	0.38	−0.29
Performance cost	−0.98	−0.82	0.72	0.71	0.70	−0.54
Performance percentage	−0.98	−0.92	0.71	0.71	0.62	−0.44
Ketosis rate	1.00	0.93	−0.74	−0.74	−0.64	−0.47

## References

[1] Drackley JK (1999). ADSA foundation scholar award: biology of dairy cows during the transition period: the final frontier?. *Journal of Dairy Science*.

[2] Herdt TH, Gerloff BJ (1999). *Ketosis. Current Veterinary Therapy, Food Animal Practice*.

[3] Radostits OM, Gay CC, Blood DC, Hinchcliff KW (2000). *Veterinary Medicine*.

[4] Sauer FD, Erfle JD, Fisher LJ (1973). Propylene glycol and glycerol as a feed additive for lactating dairy cows: an evaluation of blood metabolic parameters. *Canadian Journal of Animal Science*.

[5] Studer VA, Grummer RR, Bertics SJ, Reynolds CK (1993). Effect of prepartum propylene glycol administration on periparturient fatty liver in dairy cows. *Journal of Dairy Science*.

[6] Formigoni A, Cornil MC, Prandi A (1996). Effect of propylene glycol supplementation around parturition on milk yield, reproduction performance and some hormonal and metabolic characteristics in dairy cows. *Journal of Dairy Research*.

[7] Fronk TJ, Schultz LH, Hardie AR (1980). Effect of dry period overconditioning of subsequent metabolic disorders and performance of dairy cows. *Journal of Dairy Science*.

[8] Treacher RJ, Reid IM, Roberts CJ (1986). Effect of body condition at calving on the health and performance of dairy cows. *Journal of Animal Production*.

[9] Young JW, Smith TR (1994). Effects of nicotinamide on milk composition and production in dairy cows fed supplemental fat. *Antonio Research Fact Sheet*.

[10] Grummer RR (1993). Etiology of lipid-related metabolic disorders in periparturient dairy cows. *Journal of Dairy Science*.

[11] Minor DJ, Trower SL, Strang BD, Shaver RD, Grummer RR (1998). Effects of nonfiber carbohydrate and niacin on periparturient metabolic status and lactation of dairy cows. *Journal of Dairy Science*.

[12] Linn JG, Otterby DE (1990). An approach to solving feeding problems on dairy farms. *Compendium on Continuing Education for the Practicing Veterinarian*.

[13] Mahana B, Howard JL, Smith RA (1999). Dairy cow nutritional guidelines. *Current Veterinary Therapy, Food Animal Practice*.

[14] Markusfeld O, Galon N, Ezra E (1997). Body condition score, health, yield and fertility in dairy cows. *Veterinary Record*.

[15] Duffield T (2000). Subclinical ketosis in lactating dairy cattle. *The Veterinary Clinics of North America. Food Animal Practice*.

[16] Gillund P, Reksen O, Gröhn YT, Karlberg K (2001). Body condition related to ketosis and reproductive performance in Norwegian dairy cows. *Journal of Dairy Science*.

[17] Holcomb CS, Van Horn HH, Head HH, Hall MB, Wilcox CJ (2001). Effects of prepartum dry matter intake and forage percentage on postpartum performance of lactating dairy cows. *Journal of Dairy Science*.

[18] Goff JP, Horst RL (1997). Physiological changes at parturition and their relationship to metabolic disorders. *Journal of Dairy Science*.

[19] Pickett MM, Piepenbrink MS, Overton TR (2003). Effects of propylene glycol or fat drench on plasma metabolites, liver composition, and production of dairy cows during the periparturient period. *Journal of Dairy Science*.

[20] Hoedemaker M, Prange D, Zerbe H, Frank J, Daxenberger A, Meyer HHD (2004). Peripartal propylene glycol supplementation and metabolism, animal health, fertility, and production in dairy cows. *Journal of Dairy Science*.

[21] Bobe G, Young JW, Beitz DC (2004). Invited review: pathology, etiology, prevention, and treatment of fatty liver in dairy cows. *Journal of Dairy Science*.

[22] Overton TR, Waldron MR (2004). Nutritional management of transition dairy cows: strategies to optimize metabolic health. *Journal of Dairy Science*.

[23] Weiss WP, Gonzalo F (2006). Are your cows getting the vitamin they need?. *Advances in Dairy Technology*.

[24] Tamizrad K, Karkoodi K (2009). Effect of niacin supplementation on performance and blood parameters of Holstein cows. *South African Journal of Animal Sciences*.

[25] Didarkhah M, Jamili F Prevention from ketosis by application of different amount of niacin in Holstein cows in late and mid lactation.

[26] Ghorbani GR, Asadi Alamuti A (2003). *Advanced Management of Dairy Cow*.

[27] Mohebbi M (2005). *Metabolic Diseases of Dairy Cows, Causes, Consequences, Prevention*.

[28] Laranja da Fonseca LF, Lucci CS, Rodrigues PHM, Santos MV, Lima AP (1998). Supplementation of propylene glycolto dairy cows in periparturient period: effects on plasma concentration of BHBA, NEFA, and glucose. *Journal of Animal Science*.

[29] Stokes SR, Goff JP (2001). Evaluation of calcium propionate and propylene glycol administered into the esophagus at calving. *The Professional Animal Scientist*.

[30] Bartlett CA, Schwab CO, Smith JW, Holter JB (1983). Supplemental niacin for dairy cows under field conditions. *Journal of Dairy Science*.

[31] Rabelo, Rezende RL, Bertics SJ, Grummer RR (2003). Effects of transition diets varying in dietary energy density on lactation performance and ruminal parameters of dairy cows. *Journal of Dairy Science*.

[32] Keady TWJ, Mayne CS, Fitzpatrick DA, McCoy MA (2001). Effect of concentrate feed level in late gestation on subsequent milk yield, milk composition, and fertility of dairy cows. *Journal of Dairy Science*.

